# Renal Cell Carcinoma in Fistulizing Crohn's Disease Patient Who Received Anti-TNF *α* Therapy

**DOI:** 10.1155/2021/5593067

**Published:** 2021-04-21

**Authors:** Abdullah Mohammed Albishi, Rafaat Chakik, Mohammed Bazeed

**Affiliations:** ^1^Gastroenterology and Endoscopy Department, Armed Forces Hospital Southern Region, Khamis Mushayt, Saudi Arabia; ^2^Radiology Department, Armed Forces Hospital Southern Region, Khamis Mushayt, Saudi Arabia

## Abstract

Inflammatory bowel diseases are chronic inflammatory diseases affecting the gastrointestinal tract with different clinical presentations. These chronic inflammatory diseases are associated with an increased risk for both intestinal and different types of extra-intestinal malignancies. In this case report, we describe the condition of a 29-year-old Saudi male diagnosed with fistulizing ileal Crohn's disease 7 years ago. The patient presented to the gastroenterology clinic with left flank pain for the last 2 months, which started gradually. The pain was dull, intermittent, and without a history of fever, dysuria, or hematuria. The patient was passing 3-4 times bowel motion, watery without blood or mucus. On examination, the patient looked well. Abdomen examination revealed a soft and lax abdomen with no tenderness or organomegaly. CT  abdomen showed a well-defined hypodense focal lesion originally from the left kidney near the hilum region with a clue sign. Colonoscopy was performed and showed only terminal ileitis. The patient was referred to a urologist for further action. The patient was seen by the urologist, and they are planning for partial left nephrectomy. The renal surgical specimen histopathology was reported later as renal cell carcinoma.

## 1. Introduction

Renal cell carcinoma (RCC) originates in the lining of the proximal tubule in the kidneys. RCC is considered the commonest type of kidney malignancy in adults, and it is around 90–95% of all cases diagnosed as kidney cancers [1–3]. RCC is usually an asymptomatic disease, so the cases of RCC often have advanced course by the time of diagnosis [4]. Symptoms of RCC are different, but often include blood in the urine which is around 40% of the cases at the time they first seek medical attention, flank pain (40%), a mass in the abdomen or flank (25%), weight loss (33%), high blood pressure (20%), and fatigue [5]. The first step needed to diagnose RCC is a combination of patient symptoms, signs, and checking for any risk factors for RCC development. Based on the symptoms and signs presented, a range of laboratory tests (using blood and/or urine samples) may also be considered as an initial screening process to provide sufficient quantitative analysis of any abnormalities in electrolytes, renal and liver function, and blood clotting times. On physical examination, the abdomen palpation may reveal the presence of a mass or organ enlargement [6]. The main diagnostic tools for detecting RCC include ultrasound, computed tomography (CT), and magnetic resonance imaging (MRI) of the kidneys [7].

Chronic inflammation as well as drug-induced immunosuppression associated with inflammatory bowel disease especially Crohn's diseases particularly immunosuppressive medications such as thiopurines and methotrexate may play an important role in the development of different types of extra-intestinal malignancies by inducing DNA damage or impairing immunosurveillance of tumor cells [8, 9].

## 2. Case Presentation

A 29-year-old Saudi male was diagnosed with fistulizing ileal Crohn's disease 7 years ago and received adalimumab 40 mg subcutaneous every 2 weeks since being diagnosed. The patient was presented to the gastroenterology clinic for follow-up. He was complaining of left flank pain for the last 2 months, which started gradually. The pain was dull, intermittent, and without a history of fever, dysuria, or hematuria. The patient was passing 3-4 times bowel motion, watery without blood or mucus. He had no history of skin rash, joint pain, eye pain, redness, or jaundice and no history of NSAID use or recent intake of antibiotics.

Family history similar condition was negative. The patient gave a history of perianal complex fistulae which were managed surgically before diagnosed with Crohn's disease. The last colonoscopy showed terminal ileitis.

## 3. Clinical Examination

The patient looked well, with no pallor, no jaundice, or lymphadenopathy. The patient was vitally stable, afebrile. Cardiac and chest examinations were free. Abdomen examination revealed a soft and lax abdomen with no tenderness or organomegaly. Left and right renal angles were normal with no tenderness and no palpable masses. PR examination was normal with no obvious fistulae or hemorrhoids.

## 4. Investigation

WBC = 11, Hb = 15.2 g/dl, PLT = 320, and INR = 1.0.

Liver function tests and renal profile were normal. CRP = 14.5.

Urine analysis: normal, no nitrate, no WBC or RBC, and no cast.

### 4.1. Imaging

**Figure 1:**
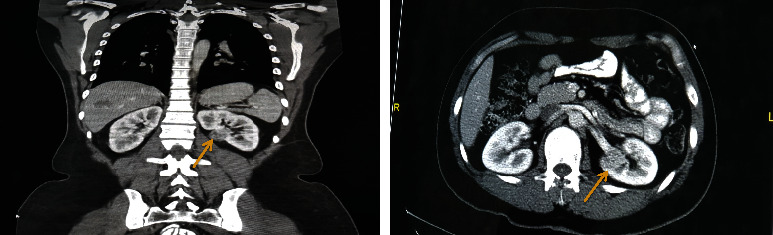
Axial and coronal view of abdominal CT scan showed a well-defined hypodense focal lesion originally from left kidney near hilum region with clue sign. The renal lesion showed inhomogeneous enhancement in the arterial phase with partial wash out in delayed phase, and it measured 2.6 cm × 2.6 cm.

CT  abdomen was performed and showed well-defined hypodense focal lesion originally from the left kidney near hilum region with clue sign. The renal lesion shows inhomogeneous enhancement in the arterial phase with partial wash out in delayed phase, it measured 2,6 cm × 2,6 cm, to rule out the possibility of renal cell carcinoma as shown in Figure 1.

Colonoscopy was performed and showed normal colonic mucosa up to terminal ileum. Terminal ileum biopsy showed acute on top of chronic ileitis.

The patient was referred to a urologist for further action. The patient was seen by the urologist, and they are planning for partial left nephrectomy. The surgical specimen histopathology was reported later as renal cell carcinoma.

### 4.2. Treatment

The patient has fistulizing ileal Crohn's disease and he needed treatment definitely, but a continuation on anti-TNF was not advised due to a history of malignancy. The patient started on anti-integrin medication (vedolizumab).

### 4.3. Outcome and Follow-Up

Follow-up after 6 months revealed that the patient was doing well with no abdominal pain. He was passing 2-3 times bowel motion. No extra-intestinal manifestation was observed. CBC, calprotectin, and CRP were normal. A follow-up CT  of the abdomen was normal. The patient was advised to continue current medications with regular follow-up.

## 5. Discussion

**Figure 2:**
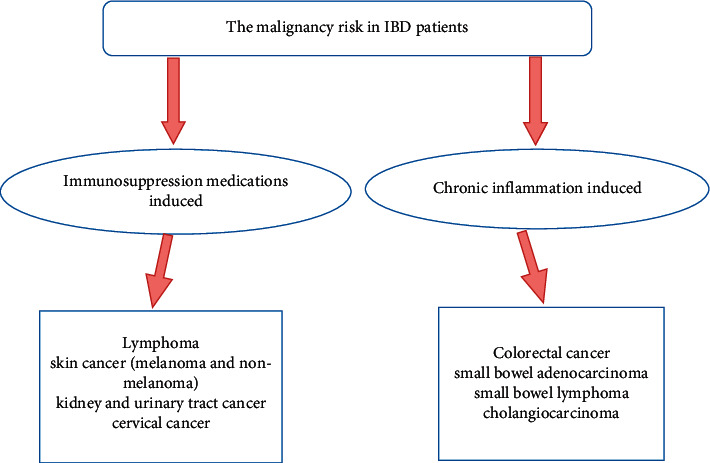
The risk of malignancies in IBD patients and related factors.

Inflammatory bowel diseases (IBDs), including Crohn's disease (CD), ulcerative colitis (UC), and indeterminate colitis, are chronic inflammatory diseases affecting the gastrointestinal tract with different various clinical presentations. These kinds of chronic inflammatory diseases are associated with an increased risk for both intestinal and different extra-intestinal malignancies as well [10, 11]. The malignancy risk in IBD patients is mainly related to chronic inflammation and/or immunosuppression medications induced as shown in Figure 2 [12]. Especially immunosuppressive drugs may play an important role in the development of different types of extra-intestinal malignancies by inducing DNA damages or impairing immunosurveillance of tumor cells [13, 14]. The potential associated risk of malignancy is an important growing concern given the need for prolonged immunosuppressive therapy in IBD patients, especially given the aging IBD population.

The current case report was done to add more evidence for the association between Crohn's disease as one of the inflammatory bowel disorders (IBD) and renal malignancies. The association between renal cell carcinoma and IBD was reported in a cohort study including 180 patients [15]. However, the association with multilocular cystic renal neoplasm (MCRN) of low malignant potential has not been described previously. Soumaya et al. [16] reported that a patient with Crohn's disease and incidental multilocular cystic renal neoplasm (MCRN) of low malignant potential who presented with complicated Crohn's disease with the deep collection was treated with antibiotic including cefotaxime and metronidazole. After 2 weeks of treatment, the patient underwent a renal lumpectomy. The specimen gross examination revealed a well encapsulated, multiseptate tumor mass of 2.5 cm.

Wauters et al. [17] conducted a retrospective case-control and cohort study to detect the risk of renal cell carcinoma (RCC) with anti-tumor necrosis factor (anti-TNF) therapy in inflammatory bowel disease (IBD) and rheumatic diseases. The study revealed that RCC was confirmed in seven anti-TNF-exposed (TNF+) and 21 anti-TNF-naive (TNF-) IBD and one TNF+ and 26 TNF- RD patients.

The main differential diagnosis of a renal mass in a patient with Crohn's disease is pyelonephritis, renal malignancy, and inflammatory pseudotumor. Pyelonephritis can be caused by either bacterial or fungal infections due to the effect of immunosuppressive treatments. Imaging features such as enlargement of the kidney, perinephric stranding, and collection or abscess formation may be an indicator to have a biopsy [18].  The involvement of perinephric space, renal vein invasion, and metastases are gold patterns that may differentiate renal tumor which is confirmed by histology [19]. Renal inflammatory pseudotumor may be also considered as the main presentation for Wegener's granulomatosis in cases with Crohn's disease [20].

A considerable portion incidentally had asymptomatic and low-stage tumors in patients with inflammatory bowel disease who require periodic abdominal imaging at a relatively young age especially patients on TNF treatment as well as rheumatic and general populations. However, potential treatment-related or disease-related risks cannot be excluded as patients with severe disease courses are more likely to receive concomitant immunosuppressive therapy.

Use of thiopurines carries a high rate of risk of kidney and bladder cancers in transplant recipients as well as the IBD patients; although the risk is almost restricted to older smoker males, no such increase was seen in patients who use anti-TNF [21].

Once the malignancy is diagnosed in IBD patients with biologics, the risk of IBD exacerbation if medication is stopped, versus the risk of progression of the malignancy if medication is continued, needs to be balanced. If a localized malignancy can be resected by the surgery, the immunosuppressive therapy could be maintained, but biologics should be stopped if chemotherapy will be started for the treatment of a solid tumor as chemotherapy which has an immunosuppressive effect and can induce and maintain IBD remission [22].

The treatment of IBD with biologics is not totally contraindicated in a patient with past history of malignancy, and decisions should be individualised. A delay of at least 2 years after successful malignancy eradication is appropriate, but for those malignancies with a high risk of late metastatic spread (including breast, malignant melanoma, and renal cell carcinoma) this should be extended to 5 years [23].

ECCO Guideline/Consensus Paper demonstrates that there is currently no evidence that the overall risk of malignancy is increased in patients being treated with anti-TNF agents alone [24].

## 6. Conclusion

Our case report focused on IBD as a risk factor to develop renal complications including renal cell carcinoma. IBD patients who are diagnosed with RCC are usually at a younger age as well as at an earlier stage of the disease. Immunosuppressive medications and anti-TNF*α* therapy may be the contributing agents and not adversely affect the survival. Patients if treated properly may show clinical improvement after treatment modifications. Periodic abdominal examination and imaging are required for IBD patients to help in the early diagnosis of any extra-intestinal complications with better survival.

## Data Availability

All data are available from the corresponding author upon request.
